# Ca^2+^/calmodulin-dependent Protein Kinases in Leukemia Development

**DOI:** 10.33696/immunology.3.091

**Published:** 2021

**Authors:** Changhao Cui, Chen Wang, Min Cao, Xunlei Kang

**Affiliations:** 1 Center for Precision Medicine, Department of Medicine, University of Missouri, 1 Hospital Drive, Columbia, Missouri 65212, USA; 2 School of Life Science and Medicine, Dalian University of Technology, Liaoning 124221, China

**Keywords:** CaMKII, CaMKI, CaMKIV, Leukemia, ITIM containing receptor, Signaling pathway, Therapeutic target

## Abstract

Ca^2+^/calmodulin (CaM) signaling is important for a wide range of cellular functions. It is not surprised the role of this signaling has been recognized in tumor progressions, such as proliferation, invasion, and migration. However, its role in leukemia has not been well appreciated. The multifunctional Ca^2+^/CaM-dependent protein kinases (CaMKs) are critical intermediates of this signaling and play key roles in cancer development. The most investigated CaMKs in leukemia, especially myeloid leukemia, are CaMKI, CaMKII, and CaMKIV. The function and mechanism of these kinases in leukemia development are summarized in this study.

## Introduction

Calcium (Ca^2+^) is an intracellular universal second messenger that regulates a variety of cellular processes. Many biological processes, including gene transcription, cell cycle, migration, and apoptosis, are affected by changes in intracellular Ca^2+^ signaling [1,2]. Disruption of normal Ca^2+^ signaling can cause tumorigenic phenotypes [3].

Ca^2+^ signaling works by forming a complex with calmodulin (CaM), a 148-amino-acid protein that transduces signals in response to intracellular Ca^2+^ elevation. Ca^2+^ binding significantly alters CaM’s conformation and enhances its affinity for a wide range of CaM-binding proteins. Since the discovery of CaM in 1970 as a Ca^2+^ regulator, there have been over 80 Ca^2+^/CaM-regulated protein kinases described [4]. However, based on their substrate specificity, not all CaM-regulated kinases are Ca^2+^/CaM-dependent protein kinases (CaMKs). For example, the once called CaMKIII is now termed eukaryotic elongation factor 2 (eEF2) kinase, due to containing a small number of substrates [5].

## Structure and Activation

**Figure 1:**
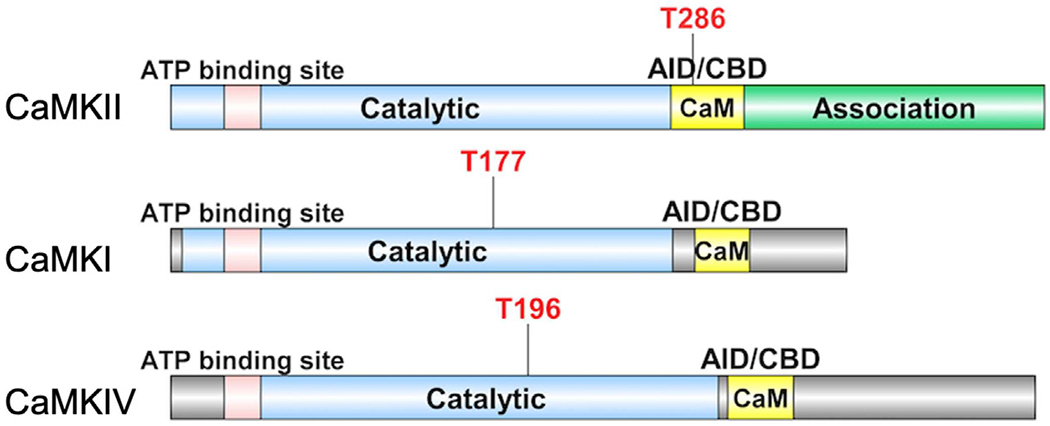
Schematic diagrams of CaMKII, CaMKI, and CaMKIV. The subunit structures with key residues involved in their regulation by phosphorylation (red font). See text for details.

The classic CaMKs include CaMKI, CaMKII, and CaMKIV, each of which has multiple isoforms. They are multifunctional serine/threonine protein kinases that regulate the development and activity of different kinds of cell types through a variety of substrates [6–8]. The structure of CaMKs is critical for their activation and regulation (Figure 1). Similarly, they all have an N-terminal kinase domain, followed by a regulatory domain (consisting of Ca^2+^/CaM binding domain (CBD) and auto-inhibitory domain (AID)). The ADP/ATP binding site locates between the small and large lobes of CaMKs’ kinase domains. CaMKII, on the other hand, has a special self-association domain at the C-terminus that allows it to form holoenzymes [8–10].

CaMKs have a special phosphorylation-dependent mechanism for the regulation of kinase activity.

The changes in intracellular Ca^2+^ concentration trigger Ca^2+^ binding to the ubiquitously expressed CaM induces a conformational transition, which sparks its binding to CBD of CaMKs. The adjacent AID will then be released, triggering CaMK activation. The difference is, for CaMKII, the Thr-286 residue in the regulatory domain is autophosphorylated before kinase activation. While CaMKI/CaMKIV is phosphorylated by an upstream kinase, CaMKK at Thr-177 and Thr-196 residue, respectively, both located in the kinase domain. Despite the fact that CAMKK activates both CaMKI and CaMKIV, their distinct subcellular distributions after phosphorylation, cytosolic for CaMKI versus nuclear localization for CaMKIV, can allow them to play different roles in distinct cellular settings [9,11].

## CaMKs in Cancer Development

Effective cell migration, which is critical for cancer metastasis, requires proper Ca^2+^ signaling control. Many CaMKs including CaMKI, CaMKII, and CaMKIV play a role in cell-migration-related cytoskeleton dynamics. Thus, the role of CaMKs in tumor cell invasiveness and metastatic potential is well implicated [3,12].

Iwatsubo’s group found that store-operated Ca^2+^ entry regulates melanoma proliferation and cell migration by activating CaMKII. Further, they demonstrated that CaMKII inhibition suppressed MAPK signaling pathway, which can inhibit human melanoma cell migration and metastasis in the lungs [3,13,14]. Interestingly, the CaMKII/MAPK signaling axis was also linked to colon cancer, inhibiting CaMKII decreased cancer cell proliferation, migration, and invasion [15]. The CaMKK pathway has been shown to promote cerebellar granule precursor migration and differentiation during normal cerebellar development via CaMKIV [16], while CaMKK/CaMKI cascade regulates basal medulloblastoma cell migration via Rac1. In addition, pharmacological CaMKK inhibition blocks both estrogen-induced Rac1 activation and medulloblastoma migration [17]. These findings indicate that the differential regulation of CaMKs in normal and malignant scenarios is context-dependent. McDonnell’s group found that CaMMKβ is highly expressed in the prostate and is further elevated in prostate cancers. Using cellular models of prostate cancer, they demonstrated that CaMKK/AMPK regulates androgen-dependent migration of prostate cancer cells [18]. All these studies indicate CaMKs may be involved in different signaling pathways to manipulate cancer development.

By using a variety of CaM antagonists or CaMKs specific inhibitors, the researchers have corroborated the roles of CaMKs in a multitude of tumor types, which can prevent cell growth, invasiveness, and /or metastasis [19–24]. For detail reviews on the role of CaMKs in cell migration and cancer metastasis, see [3,25–27].

## Relevance to Leukemia

In comparison to a large number of studies on CaMKs in neurology and solid tumors, research on CaMKs’ function in controlling hematopoiesis and leukemia has been scarce. Leukemia diseases are not classified as metastatic cancer. Because they are thought to already be widespread when they are diagnosed. Below, we discuss the role of CaMKs in leukemia development based on work of ours and others. Emphasis is given to CaMK’s function in myeloid leukemia, as well as the signaling pathway they are involved.

Using the TCGA database of AML patients, we conducted an *in silico* study of the relationship between gene expression and overall survival in AML patients. (https://tcga-data.nci.nih.gov/tcga/). The expression of most *CAMKs* analyzed showed a negative correlation between expression and patient survival, which including *CAMKI, CAMK2A, CAMK2D, CAMK4, CAMKK2* [28,29]. These results suggest that many CaMKs directly support human leukemia cell growth. Here, we summarize the leukemia-related roles of individual CaMKs.

## CaMKII

CaMKII is the most widely studied CaMKs, which consists of four homologous (CaMKIIa, CaMKIIb, CaMKIIg, and CaMKIId) [1]. CaMKII’s autophosphorylation is one of its most important functional characteristics which means that its activation is self-contained and less influenced by Ca^2+^ concentration or calmodulin regulation. CaMKII makes up about 1% to 2% of total brain protein, and numerous studies have shown that it plays an important role in controlling neuronal cell growth and function [26].

Although aberrant activation of CAMKII has been linked to different hematopoietic malignancies [30,31], most studies focus on one of its isoforms, CaMKIIg, and its role in myeloid leukemia. CaMKIIg is preferentially expressed in myeloid cells [32,33]. Furthermore, the activation of CaMKIIg is greatly increased in leukemia stem/progenitor cells but not in normal hematopoietic cells [34–36]. By phosphorylating and inhibiting the transcriptional function of retinoic acid receptors (RARs), CaMKIIg prevents the differentiation of myeloid leukemia cells. Coordinately, CaMKII inhibitors improve the differentiation of myeloid leukemia cells [32]. This finding was further extended to non-retinoic acid responsive myeloid leukemia cells, which demonstrates that CaMKIIg plays a critical and central role in controlling the proliferation of a broad range of myeloid leukemia cells [33]. The mechanistic studies showed that CaMKII activation induced by different stress contributes to mitogenic signaling and promotes the proliferation of leukemia cells [14,37]. Strikingly, Huang’s group proved that CaMKIIg is a key regulator of leukemia stem cells in chronic myeloid leukemia (CML) [35]. The findings in mouse CML leukemia model and human CML patients indicate that CaMKIIg could be a critical regulator in the progression of CML blast crisis, and they reveal a novel mechanism by which CaMKIIg promotes leukemia stem cell (LSC) self-renewal by inhibiting nuclear p27Kip1 and reawakening dormant LSCs [34,38]. The signaling studies in both AML and CML revealed that CaMKIIg acts as an important regulator of multiple cancer-related signaling pathways including NF-κB, Wnt/b-catenin, p42/p44/MAPK, and Stat3/5 networks [32,35]. The supportive role of CaMKIIg in the development and progression of myeloid leukemia was verified by the treatment of specific inhibitors or by depletion of CaMKIIg [14,32,33,35–37]. In summary, inhibition of CaMKIIg activity may be beneficial in the treatment of myeloid leukemia.

## CaMKI

Similar to CaMKII, CaMKI is multifunctional kinases that have been linked to neuronal plasticity and gene regulation [9]. In human, the CaMKI family consists of four members, each of which is encoded by a different gene: *CAMK1, PNCK, CAMK1G and CAMK1D*, which generate CaMKIa, CaMKIb, CaMKIg, and CaMKId, respectively [39].

It is reported that the isoforms of CaMKI are ubiquitously expressed at low levels [40], and expressed at high levels in several brain regions [41,42]. Interestingly, we found that CaMKI protein is well detected in both normal hematopoietic cells and AML cells. An *in silico* analysis of human *CAMK1* mRNA expression in 43 human AML samples showed that it is highly expressed in M5 AML cells [28,43]. Moreover, CaMKI is greatly expressed in AML LSCs in mouse MLL-AF9 model. Gain-of-function and loss-of-function analyses of CaMKI in AML cells *in vivo* proved that CaMKI is essential for the growth of human and mouse AML cells [28,29]. The mechanistic studies indicated CaMKI participates in the Inhibitory leukocyte immunoglobulin-like (ITIM) receptors signaling axis in leukemia development by the recruitment of SH2 domain-containing phosphatase1 (SHP-1). The activated CaMKI can be transported into the nucleus [44] and activate the downstream transcription factor cyclic AMP element-binding protein (CREB) in AML cells [29]. CREB is one of the well-known targets of CaMKs in hematopoietic cells, which can be phosphorylated and activated by CaMKs [45,46]. This novel signaling pathway identified in AML stem cells may represent a target for AML treatment [47].

## CaMKIV

CaMKIV is encoded by the *CAMK4* gene. Alternative processing yields two distinct isoforms (CaMKIVa and CaMKIVb) [48]. The CaMKIV expression pattern is similar to other CaMKs, with primarily being expressed in the brain, however, CaMKIV is also present in hematopoietic cells, testes and ovaries [49–52]. CaMKIV has been implicated in the regulation of homeostatic plasticity, neurite outgrowth, fear memory, immune and inflammatory responses [1,53].

CaMKIV have been shown to play a significant role in hematopoietic physiology and pathology in a few studies. CaMKIV/CREB/BCL-2 signaling is required for hematopoietic stem cell (HSC) activity. CaMKIV is correlated with increased apoptosis and proliferation of HSCs *in vivo* and *in vitro* [50]. We also found that, in human cord blood CD34^+^ cells, Angptl binding to ITIM receptors can trigger phosphorylation of CaMKIV [54]. CaMKIV is also a key player in the imbalance between Treg and Th17 cells. CaMKIV contributes to the reduction of IL-2 and the restriction of Treg cells in patients with SLE [55]. Since IL-2 has been shown to inhibit Th17 differentiation, CaMK4 inhibition possibly promotes Th17 differentiation indirectly [56,57]. Although HSCs in *CAMKIV*-knockout mice showed only mild defects [50], The loss of function of LILRB2/PirB or CaMKIV, on the other hand, is detrimental to AML growth. Importantly, inhibiting CaMKIV kinase activity or deleting CaMKIV significantly reduced AML stem cell activity. We discovered that phosphorylation of CREB is important downstream of CaMKIV signaling in AML cells using a rescue assay. Our findings showed that CaMKIV signaling promotes AML cell self-renewal and inhibits apoptosis [28]. As a result, inhibiting CaMKIV is likely to be successful in treating leukemia with limited hematopoietic system side effects.

## Concluding Remarks and Perspectives

Targeted therapy is an effective cancer treatment technique that has seen a lot of success in clinical trials [58]. Currently, many of the clinically used targeted cancer drugs are tyrosine kinase inhibitors. However, even though these medications have extraordinary effectiveness at first, drug resistance emerges later, limiting their utility [59]. Finding new drug targets and developing new targeted anticancer agents have accordingly become urgent for drug discovery and development. Because the different structure between tyrosine kinases and serine-threonine kinases, drugs that target CaMKs are less likely to modulate conventional kinases and, in theory, do not have or trigger cross-resistance with traditional kinase drugs. The CaMK family members, particularly CaMKII, CaMKI, CaMKIV, are appealing anti-cancer targets because they are overexpressed in myeloid leukemia as compared to normal blood cells and play a critical role in leukemia cell proliferation, differentiation and self-renewal.

In contrast to studies in other forms of tumors, there has been very little research into the role of CaMKs in leukemia. Our review reveals that most of these limited studies focus on myeloid leukemia diseases, which may be due to the relative high expression of *CAMKs* in myeloid cell lineage. Because inhibition of the expression and/or activity of CaMKs directly blocks leukemia cell growth and activity, and suppresses tumorigenesis and leukemia development, but does not much disturb normal development, CaMKs may represent ideal targets for treating leukemia. Since there are many different CaMKs, which can express differently in diverse leukemia types or subtypes. It is fascinated to see how these CaMKs interact individually and in combination in cancer cells. For example, in leukemia cell lines (U937), inhibiting CaMKII activity causes an upregulation of CaMKIV mRNA and protein. CaMKIV expression, on the other hand, inhibits CaMKII autophosphorylation and activation, as well as G0/G1 cell cycle arrest, which inhibits cell proliferation [38]. This data indicates that CaMKII suppresses CaMKIV expression to promote leukemia cell proliferation.

**Figure 2:**
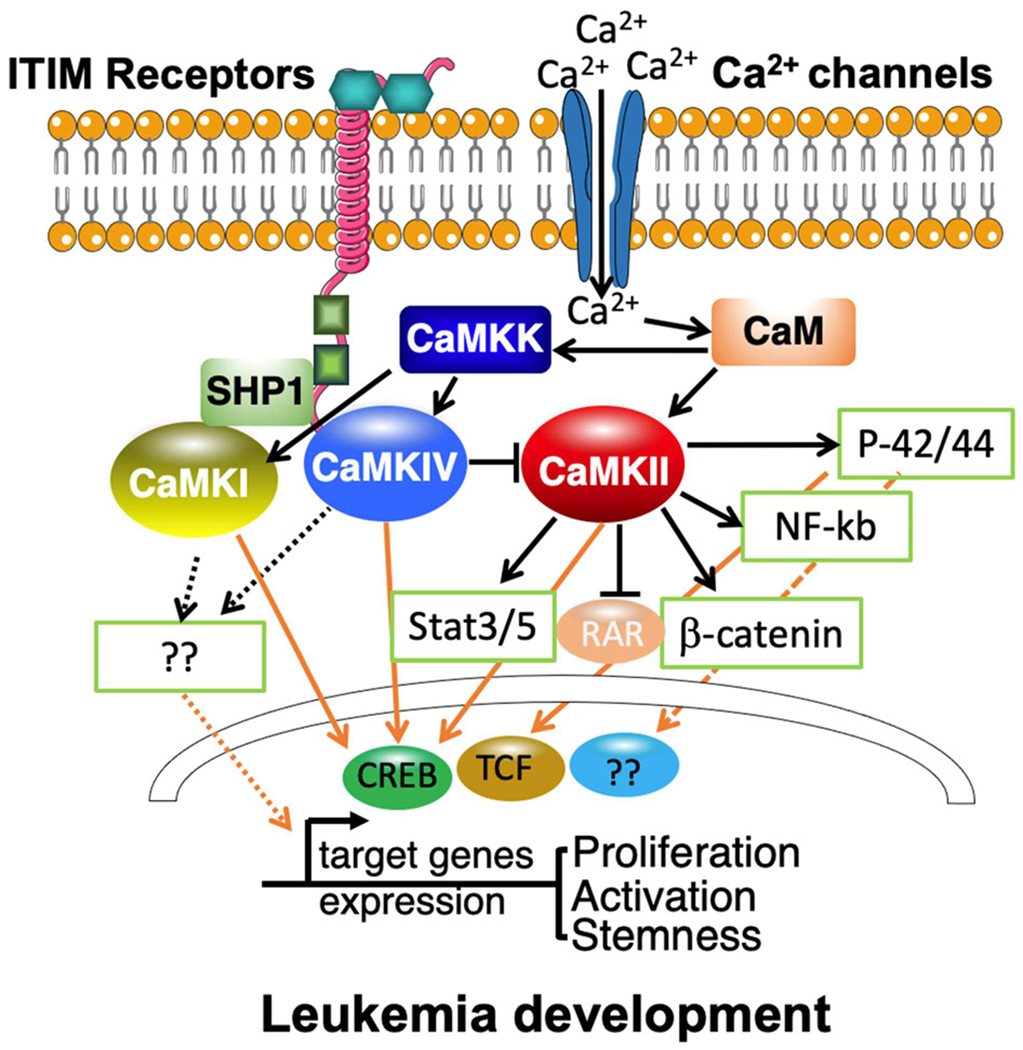
Both calcium channel signaling and ITIM containing receptor signaling are involved in leukemia progression by binding to and activating the Ca^2+^/calmodulin dependent protein kinase family. Intracellular Ca^2+^ and CaM level is upregulated in leukemia cells. The activated CaMKII will further activate different oncogenic signaling in leukemia cells, such as p42/44, NF-kB, Stat3/5, b-catenin. The signaling will translocate to the nuclear by their downstream transcription factors, such as TCF, CREB. CaMKII has also been reported to inhibit the activation of RAR to block the differentiation. ITIM containing receptors can recruit CaMKI/IV directly or indirectly by phosphatase SHP-1. CaMKI/IV can be activated by classical CaMKK pathway. The coordinated regulation of CaMKI/IV by Ca^2+^ signaling and ITIM containing receptor signaling is unknown. The activated CaMKI/IV then induce phosphorylation of the transcription factor CREB. Finally, these signaling pathway will activate downstream genes to initiate the regulation of self-renewal, survival and differentiation of leukemia cells.

Blocking CaMKs signaling in conjunction with standard therapies may be an effective method for destroying leukemia cells. In light of the tremendous importance of CaMKs signaling in leukemia development, it will be critically important to identify more components involved in this signaling (Figure 2). Besides ITIM containing receptors, whether there are any other upstream signaling involved? Our studies have identified CREB as the major downstream signaling that are activated by CaMKI/IV. If there is another transcriptional factor that is crucial for leukemia development and controlled by CaMKI and/or CaMKIV need to be further investigated. For CaMKIIg, how consequential phosphorylation of several target substrates dynamically cooperates to modulate leukemia cell proliferation or activity. These future directions may help further to clarify the role of CaMKs in leukemia development.
